# Oxidative Stress and Lipid Accumulation Augments Cell Death in LDLR-Deficient RPE Cells and *Ldlr* ^−/−^ Mice

**DOI:** 10.3390/cells12010043

**Published:** 2022-12-22

**Authors:** Parameswaran Gangadharan Sreekumar, Feng Su, Christine Spee, Eduardo Araujo, Steven Nusinowitz, Srinivasa T Reddy, Ram Kannan

**Affiliations:** 1 Doheny Eye Institute, Pasadena, CA 91103, USA; 2 Department of Molecular and Medical Pharmacology, David Geffen School of Medicine, University of California at Los Angeles, Los Angeles, CA 90095, USA; 3 Jules Stein Eye Institute, David Geffen School of Medicine, University of California at Los Angeles, Los Angeles, CA 90095, USA

**Keywords:** geographic atrophy, low-density lipoprotein receptor, oxidative stress, inflammation, retinal pigment epithelium, retinal degeneration, retinal function

## Abstract

Lipid peroxidation from oxidative stress is considered a major contributor to age-related macular degeneration (AMD). The retina is abundant with circulating low-density lipoproteins (LDL), which are taken up by LDL receptor (LDLR) in the RPE and Müller cells. The purpose of this study is to investigate the role of LDLR in the NaIO_3_-induced model of dry AMD. Confluent primary human RPE (hRPE) and LDLR-silenced ARPE-19 cells were stressed with 150 µM tert-butyl hydroperoxide (tBH) and caspase 3/7 activation was determined. WT and *Ldlr* ^−/−^ mice were administered NaIO_3_ (20 mg/kg) intravenously. On day 7, fundus imaging, OCT, ERG, and retinal thickness were measured. Histology, TUNEL, cleaved caspase 3 and lipid accumulation were assessed. Treatment of hRPE with tBH markedly decreased LDLR expression. Caspase 3/7 activation was significantly increased in LDLR-silenced ARPE-19 cells treated with tBH. In *Ldlr* ^−/−^ mice, NaIO_3_ administration resulted in significant (a) retinal thinning, (b) compromised photoreceptor function, (c) increased percentage of cleaved caspase 3 positive and apoptotic cells, and (d) increased lipid droplet accumulation in the RPE, Bruch membrane, choroid, and sclera, compared to WT mice. Our findings imply that LDLR loss leads to lipid accumulation and impaired retinal function, which may contribute to the development of AMD.

## 1. Introduction

Age-related macular degeneration (AMD), a multifactorial neurodegenerative disease, is the leading cause for legal blindness in the world. AMD includes the wet or neovascular (nAMD) form characterized by blood vessel growth and leakage, and the geographic atrophy (dry AMD) form that results in patchy loss of the retinal pigment epithelium (RPE) and photoreceptors [1,2]. Dry AMD accounts for 85% to 90% of cases, and patients experience severe visual impairment which can progress to legal blindness [1,2]. Currently, there are no approved treatments for dry AMD [2,3]. The underlying causes of AMD include persistent inflammation, lipid accumulation, oxidative stress, and impaired extracellular matrix maintenance [3,4]. However, the molecular mechanisms associated with the degenerative processes that lead to the development and progression of AMD are not well understood.

Aging-related lipid accumulation between the inner layer of the Bruch’s membrane (BrM) and the basal lamina of the retinal pigment epithelium (RPE) results in the formation of drusen, which impairs retinal function by preventing the exchange of nutrients between the choriocapillaris and the RPE [5,6]. Esterified and unesterified cholesterol, phosphatidylcholine, and other lipids make up at least 40% of the volume of drusen [5,7]. The ubiquitously expressed membrane low density lipoprotein receptor (LDLR) functions to facilitate the endocytosis of circulating LDL. Intake of lipoproteins from the bloodstream via the LDLR or phagocytosis of photoreceptor outer segments are the two major pathways that the RPE cell accumulates cholesterol [3].

Epidemiological studies showed a connection between atherosclerosis and AMD [8,9,10,11]. Extracellular lipid accumulation, oxidative stress, and inflammation are hallmarks of both diseases. The genetic deletion of *Ldlr* in mice raises cholesterol levels on the chow diet to 200–300 mg/dL and has been extensively used as an established murine model of atherosclerosis [12]. *Ldlr* ^−/−^ mice on a Western diet have plasma lipoprotein profiles similar to that of hypercholesteremic human plasma samples [13]. Therefore, *Ldlr* ^−/−^ mice provide a unique opportunity to test whether LDLR modifies lipid metabolism and impairs retinal function.

The role of LDLR in an atrophic model of AMD has not been studied to date. Previous work showed that LDLR deficiency caused the accumulation of membrane-bound translucent particles in the BrM, along with increased vascular endothelial growth factor (VEGF) expression when fed with a high-fat diet [10,11,12]. We hypothesized that LDLR deficiency would augment RPE cell death with oxidative stress and cause lipid accumulation and impaired retinal function in a mouse model of dry AMD. Therefore, we investigated retinal function, differences in fundus, OCT, and histology, apoptosis, and neutral lipid accumulation in *Ldlr* ^−/−^ mice and control mice in the NaIO_3_-induced model of dry AMD. Moreover, we examined the effect of NaIO_3_-induced oxidative stress on LDLR protein expression and regulation in hRPE cells.

## 2. Materials and Methods

### 2.1. Retinal Pigment Epithelial Cell Culture

All procedures adhered to the tenets of the Declaration of Helsinki for research involving human subjects. The RPE cells were isolated from human fetal eyes received from Advanced Bioscience Resources Inc. (Alameda, CA, USA) and Novogenix Laboratories, LLC (Los Angeles, CA, USA) and cultured as previously described [14]. Confluent cell cultures between passages 2 and 4 were used in all experiments. In brief, the hRPE cells were grown in Dulbecco’s modified Eagle medium (DMEM, Gibco) with 10% fetal bovine serum (FBS, Laguna Scientific, Laguna Niguel, CA, USA). Highly differentiated fetal human RPE cells were cultured on Transwell filters as described earlier [15].

### 2.2. Localization of LDLR in fhRPE Cells with and without Oxidative Stress

Localization of LDLR was performed on confluent RPE cells grown in four-well chamber slides. Confluent cells were treated with 150 μM tert-Butyl hydroperoxide (tBH; Sigma-Aldrich Corp., St. Louis, MO, USA) for 24 h in serum-free culture medium [16,17]. In this and other subsequent in vitro studies, we used serum-free conditions since fetal bovine serum contains low density (LDL) lipoproteins and LDL is a major lipoprotein component. Upon completion of the experiment, cells were fixed with 4% paraformaldehyde (PFA) for 20 min, then permeabilized in 0.2% Triton X-100 for 5 min, followed with blocking in Animal-Free Blocker (SP-5035-100, Vector Lab, Newark, CA, USA) for 30 min. Cells were incubated overnight with 1:100 dilution of LDLR antibody (# PA5-22976, Invitrogen, Carlsbad, CA, USA) and for 30 min with FITC-labeled secondary antibodies. Samples were viewed under a laser scanning confocal microscope (LSM 710, Carl Zeiss, Thornwood, NY, USA). Mean fluorescence intensity was quantified using image J [18,19]. In separate studies, we also examined polarized localization of LDLR in RPE monolayers.

### 2.3. siRNA-Mediated Knockdown of LDLR and Caspase-3/7 Activation Using IncuCyte Cell Apoptosis Assay

ARPE-19 cells (passage # 10, ATCC, Manassas, VA, USA) were validated by RT-PCR evidence for the expression of Keratin-18, BEST1 and CRALBP (Figure S1). The primers used are shown in the Supplemental Table S1. ARPE-19 cells at 40–50% confluence were used for all transfection studies. The siRNA targeting human LDLR sequences (Hs-LDLR_5 FlexiTube siRNA; 5′-TTGGACAGATATCATCAACGA -3′) (Qiagen, Valencia, CA, USA) and negative control siRNA (Qiagen) were mixed with RNAi MAX transfection reagent (Life Technologies, Carlsbad, CA, USA). To avoid cytotoxicity, the transfection medium was replaced with a complete medium at 6 h after siRNA transfection. LDLR mRNA expression was analyzed by real-time RT-PCR after 24 h post-transfection. To study the effect of LDLR knockdown on caspase-3/7 activation, the LDLR silenced cells were incubated with tBH (300 µM) for 24 h and assayed for cell death in serum-free medium. Caspase-3/7 reagent SYTOX Green diluted in cell culture media (1:1000 dilution) to make a total volume of 100 μL/well. Each treatment condition was performed in 10–12 wells. Cell apoptosis was monitored for 24 h using a live cell analysis system (IncuCyte ZOOM; Essen Bioscience, Ann Arbor, MI, USA), as described earlier [20].

### 2.4. NaIO_3_-Induced RPE Degeneration Mouse Model

Male C57BL6/J mice aged six to eight weeks from The Jackson Laboratory (The Jackson Laboratory, Bar Harbor, ME, USA) were used. *Ldlr* ^−/−^ mice on a C57BL/6J background were bred from the breeding colony of the Department of Laboratory and Animal Medicine at the David Geffen School of Medicine at the University of California, Los Angeles (UCLA). All animal procedures are approved by the UCLA Institutional Animal Care and Use Committee (# ARC 2019-060). Experimental procedures used in this study were conducted in accordance with National Institutes of Health guidelines and the Association for Research in Vision and Ophthalmology Statement for the Use of Animals in Ophthalmic Vision Research.

The NaIO_3_-induced mouse model of RPE atrophy has been well characterized in our laboratory [18,21,22]. Based on our previous studies, we used a single dose of 20 mg/kg BW NaIO_3_ administered via the tail vein. Control animals received a tail vein injection of sterile PBS. Male mice (WT and *Ldlr* ^−/−^) were divided into four treatment groups, each group consisted of eight mice. Group 1: WT + PBS; Group 2: WT + NaIO_3_; Group 3: *Ldlr* ^−/−^ + PBS and Group 4: *Ldlr* ^−/−^ + NaIO_3_. All mice were maintained on a standard chow diet. Animals were euthanized on day 7 post NaIO_3_ injection.

### 2.5. Spectral Domain Optical Coherence Tomography (SD-OCT) and Fundus Imaging

Mice were anesthetized (ketamine and xylazine) and pupils were dilated with one to two drops of Tropicamide Ophthalmic Solution 1% (Bausch & Lomb, Tampa, FL, USA). To create a consistent, optically transparent interface, an ocular demulcent solution containing hydroxypropyl methylcellulose ophthalmic (Gonak, Akorn, Lake Forest, IL, USA) was applied to the eye. Ultra-high resolution spectral domain optical coherence tomography (SD-OCT) imaging was performed on both eyes at the end of experiments (Bioptigen SD-OCT system, Research Triangle Park, Durham, NC, USA) as described [21]. In short, a series of 100 b-scans were collected, stacked, and aligned spatially to form a registered three-dimensional rendering of the retinal volume. By averaging 20 separate b-scans along the same horizontal axis and spatially aligning them, a high-resolution horizontal b-scan centered on the optic nerve head was obtained. The total retinal thickness, measured as the distance (in µm) between the retinal pigment epithelium and the inner limiting membrane, was calculated for each location. The Micron II retinal imaging microscope (Phoenix Research Laboratories, Inc., CA, USA) was used to capture fundus pictures.

### 2.6. Electrophysiology

Following overnight dark adaptation and pupil dilation, ERGs were recorded from the corneal surface using a gold loop corneal electrode together with a mouth reference and tail ground electrode. To guarantee electrical contact and preserve corneal integrity, a drop of methylcellulose (2.5 percent) was applied to the corneal surface. A heated water pad was used to keep the body temperature at 38 °C. Responses were amplified (Grass CP511 AC amplifier, × 10,000) and digitized using an I/O board (National Instruments, Austin, TX, USA). Signal processing was performed with custom software LabWindows/CVI (National Instruments, Austin, TX, USA). For each stimulus intensity, responses were computer-averaged with up to 50 records averaged for the weakest signals. A signal rejection window was used to eliminate artifacts. Flash presentation frequency was set to 1 Hz except for the brightest flashes which were slowed to 0.2 Hz. For scotopic responses, intensity response functions were fitted with a standard Naka-Rushton function to estimate Vmax, the maximum saturated b-wave amplitude.

### 2.7. Retinal Histology

The posterior eye cups were embedded in OCT medium and snap-frozen in liquid nitrogen [18]. Cryostat sections (8 μm) of snap-frozen posterior eyecups were processed 7 days after NaIO_3_ challenge. After fixation with 4% paraformaldehyde for 30 min, sections were prepared for hematoxylin and eosin (H & E) staining and imaged with Aperio digital pathology slide scanner (Leica Biosystems, Buffalo Grove, IL, USA).

### 2.8. TUNEL Staining

In Situ Cell Death Detection Kit (# 12156792910, Roche, Indianapolis, IN, USA) or the terminal deoxynucleotide transferase dUTP nick end labeling (TUNEL) assay was performed on retinal sections according to the manufacturer’s instructions. Retinal sections (8 µm) were fixed in 4% paraformaldehyde (PFA), permeabilized with 0.1% Triton X-100, followed with incubation with the TUNEL reaction mixture for an hour at 37 °C, in a humid atmosphere. After washing, the label incorporated at sites of DNA damage was visualized by digital fluorescence microscope (KEYENCE, Itasca, IL, USA). TUNEL-positive cells were counted as described earlier, and data were expressed as percent of total cells undergoing apoptosis [18].

### 2.9. Immunofluorescence Staining

Retinal sections were fixed in 4% PFA and permeabilized with 0.1% Triton X-100. Samples were blocked with Animal-Free Blocker for 30 min. Sections were incubated with either rabbit polyclonal antibody against cleaved caspase-3 (1:100 dilution, #9661, Cell Signaling Tech, MA, USA) or rabbit polyclonal antibody against LDLR (1:100 dilution, # PA5-46987, Invitrogen, Carlsbad, CA, USA) or mouse monoclonal to perilipin 1 (1:100 dilution, # 390169, Santa Cruz Biotech, Dallas, TX, USA) overnight. Sections were then incubated with goat fluorescein-conjugated anti-rabbit secondary antibody for 30 min. Images were obtained using a Keyence fluorescence digital microscope. For quantification of immunofluorescence, digital images were analyzed (*n* = 5–6), and the average total corrected fluorescence (background subtracted) was calculated using ImageJ (US NIH, Bethesda, MD, USA) [18].

### 2.10. Oil Red O Staining

Retinal frozen sections (8 µm) were fixed (4% PFA, 5 min) and neutral lipids were stained with Oil Red O stain kit (#ab150678, Abcam, Waltham, MA, USA) overnight at RT. Slides were differentiated in 85% propylene glycol for 1 min, rinsed in 2 changes of distilled water, incubated in hematoxylin for 30 s, and then rinsed in tap water and 2 changes of distilled water. Images were captured with a digital microscope (KEYENCE, Itasca, IL, USA).

### 2.11. Data Analysis

Data presented are representative curves or mean ± SEM. Statistical analyses were performed by Student’s *t*-test and a one-way ANOVA, followed by Tukey’s post hoc test (GraphPad Software, La Jolla, CA). A *p* value of <0.05 was considered statistically significant.

## 3. Results

### 3.1. LDLR RPE Localization

**Figure 1:**
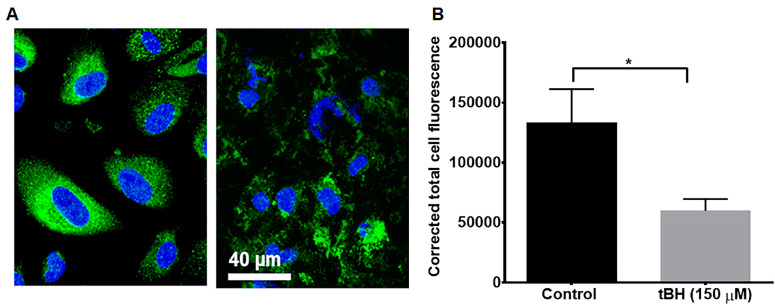
Effect of stress on the expression of LDLR in primary hRPE. (**A**). Primary hRPE cells were cultured in serum free medium and treated with 150 µM tBH for 24 h. Cells were fixed, blocked, and stained for LDLR (green). LDLR expression and localization are altered with stress. (**B)**. Quantification revealed that in the tBH-treated cells, LDLR expression was significantly lower than the control cells. Data are shown as mean ± SEM (*n* = 6, * *p* < 0.05).

In nonpolarized RPE cells, LDLR is homogeneously distributed to the cytoplasmic compartments (Figure 1A). Oxidative stress from tBH (150 µM) for 24 h caused not only altered distribution but also significantly decreased LDLR protein expression in hRPE cells (Figure 1A). To quantify the change in LDLR expression level, we measured the corrected total fluorescence of LDLR-positive cells. This method allows background subtraction and considers the area of the cell that is measured. Using this criterion, we found that LDLR expression was significantly lower in stressed hRPE cells (Figure 1B). RPE cells without primary antibody incubation served as a negative control (data not shown). Since the polarized hRPE monolayers mimic the native RPE monolayer, we also studied LDLR expression in polarized RPE cells. For this purpose, we used polarized RPE cells isolated from fetal donor eyes grown in Transwell filters for 6 weeks and verified their morphology and transepithelial electrical resistance (TER, >200 Ω·cm^2^) as in our previous studies [14]. In the polarized RPE monolayer, LDLR staining was predominantly found in the basolateral domain (Figure S2).

### 3.2. LDLR Knockdown Accelerated and Exacerbated tBH-Induced Cell Death

**Figure 2:**
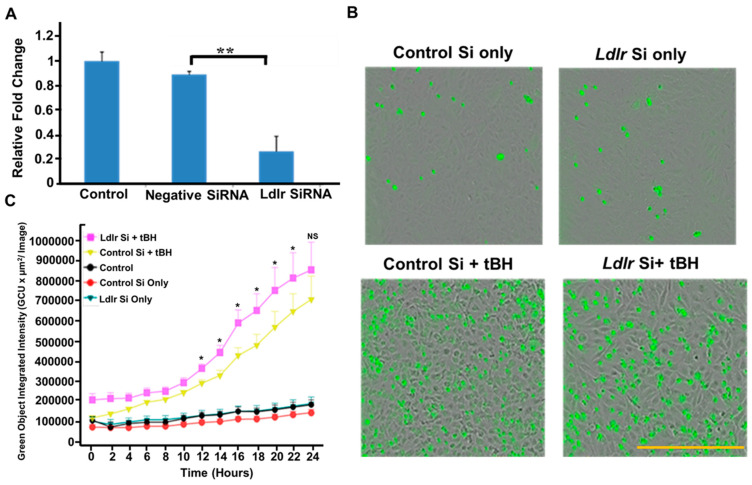
Absence of LDLR exacerbates tBH mediated caspase activation. (**A**)**.** ARPE-19 cells were transfected with negative control siRNA (10 nM) or LDLR siRNA (10 nM). (**B**). The expression of LDLR mRNA in relation to GAPDH 24 h following transfection is shown (mean ± SEM, *n* = 3, ** *p* < 0.01). Representative images of caspase-3/7 positive cells 24 h post treatment. (**C**). Quantification of the activation of caspase-3/7 (green, fluorescent staining) for up to 24 h showing the increased activation of caspase-3/7 in LDLR silenced cells challenged with tBH (300 µM). Asterisks represent comparison of fluorescence intensities of control Si + tBH and *Ldlr* Si + tBH at various time points. (mean ± SEM, *n* = 6). NS: not significant. * *p* < 0.05, scale bar: 400 µm.

It has been reported that RPE cells take up circulating LDL via LDLR mediated mechanism [23]. *Ldlr* ^−/−^ mice are not able to incorporate plasma cholesterol into cells sufficiently, resulting in increased circulating total cholesterol and subsequent cell death [24]. We next examined whether absence of LDLR alters RPE survival. To explore this, we silenced LDLR in ARPE-19 cells using siRNA and then challenged the ARPE-19 cells with oxidative stress for 24 h. The silencing efficiency was verified at the mRNA level, which showed 80% LDLR knockdown (Figure 2A). The rate and degree of cell death was measured in real-time using the IncuCyte imaging system. The caspase-3/7 reagent is a cell-permeable, non-toxic dye conjugated to the caspase-3/7 recognition and cleavage sequence (DEVD) [25]. The dye can only fluoresce green when bound to DNA, enabling the identification of dead and dying cells. Cell death is indicated by widespread cell clumping of green-fluorescing cells that have cleaved the caspase-3/7 reporter reagent (Figure 2B). Our data showed that 300 µM tBH treatment significantly increased caspase activation over time in negative control siRNA transfected ARPE-19 cells. However, when LDLR silenced cells were exposed to tBH, there was a significantly higher caspase activation at later time points except for 24 h value which did not reach significance (Figure 2C). Overall, our data suggests a protective role for LDLR in tBH-induced cell survival.

### 3.3. Deficiency of LDLR Exacerbated Retinal Degeneration In Vivo

**Figure 3:**
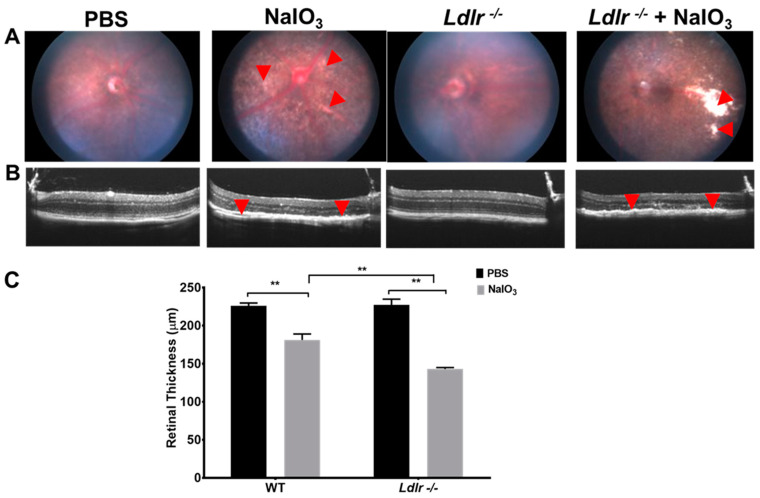
Effect of NaIO_3_ treatment on fundus and OCT in 8 wk old WT and *Ldlr* ^−/−^ mice. Mice were administered NaIO_3_ (20 mg/kg BW) and fundus (**A**), OCT (**B**,**C**) were performed on Day 7. In *Ldlr* ^−/−^ mice, retinal degeneration was more severe as evidenced by larger white patches (**A**). NaIO_3_ treatment caused significant thinning of the outer layers (red arrow heads) in WT mice which was further significantly decreased in *Ldlr* ^−/−^ mice (red arrow heads) (**B**,**C**). (mean ± SEM, *n* = 5–6; ** *p* < 0.01).

To address whether Ldlr deletion in vivo affects the retinal structure and function from oxidative stress, we used a murine model of NaIO_3_-induced acute degeneration [18,21]. We selected the dosage of 20 mg/kg NaIO_3_ based on previous studies that retinal structure, especially RPE morphology and function are significantly compromised with this dose [21]. At 7 days after NaIO_3_ administration, the number of white spots (white arrow heads), indicative of RPE degeneration, were significantly more in *Ldlr* ^−/−^ mice compared to WT mice, suggesting that Ldlr deletion renders retina more susceptible to oxidative stress (Figure 3A). Representative optical coherence tomography (OCT) images of the retina 7 days after NaIO_3_ treatment revealed a thinning of the outer layers, indicating photoreceptor loss (Figure 3B,C).

### 3.4. LDLR Deficiency and or Acute NaIO_3_ Administration Reduced Visual Performance

**Figure 4:**
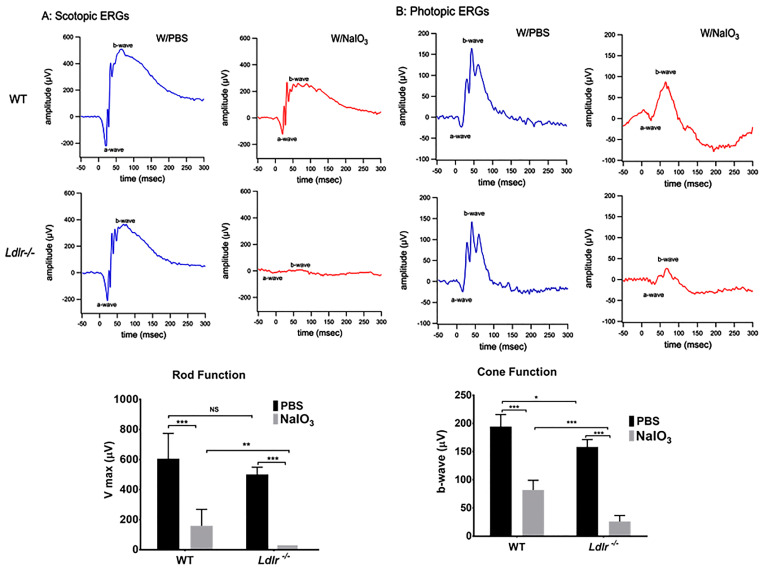
Effects of NaIO_3_ on vision function in *Ldlr* ^−/−^ and WT mice. ERG was used to study retinal function, and responses to single-intensity flashes were measured under scotopic and photopic conditions. Representative scotopic (**A**) (flash intensity = 0.02 cds/m^2^) and photopic (**B**) (flash intensity = 20.0 cds/m^2^) waveforms for the brightest stimulus from each group are shown (**upper** two panels). Averages and SEM of saturated retinal response amplitudes by group are also shown (**lower** panels). NaIO_3_ (20 mg/kg) treatment significantly reduced visual function in *Ldlr* ^−/−^ and WT mice, which was further compromised by *Ldlr* deficiency (mean ± SEM, *n* = 5–6; NS: ns: not significant, * *p* < 0.05, ** *p* < 0.01, *** *p* < 0.001).

The LDLR receptor is widely known for its role in controlling cholesterol metabolism, which is crucial for maintaining neuronal health and visual function. Therefore, we investigated whether retinal function could be compromised due to LDLR deficiency. Functional analysis by ERG under scotopic conditions revealed substantially impaired in *Ldlr* ^−/−^ mice compared to age-matched WT mice of the same age (Figure 4A). *Ldlr* ^−/−^ mice showed a decrease in photopic ERG responses as well (Figure 4B). Scotopic and photopic ERG responses in NaIO_3_-treated *Ldlr* ^−/−^ mice were almost non-detectable compared to those of matching WT mice when challenged with NaIO_3_, indicating considerable photoreceptor damage, consistent with outer retinal thinning shown by our OCT image analysis (Figure 3B,C). These data are consistent with the loss of rod and cone sensitivity in *Ldlr* ^−/−^ mice, compared with corresponding WT mice, seven days post NaIO_3_ administration.

### 3.5. Histopathological Changes and Retinal Degeneration in NaIO_3_ Administered Ldlr ^−/−^ Mice

**Figure 5:**
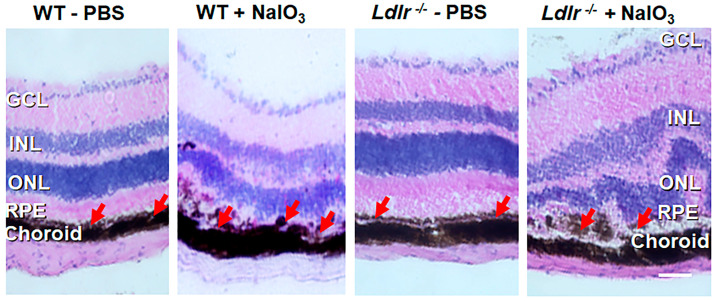
Histopathology of the retina from PBS and NaIO_3_-treated WT and *Ldlr* ^−/−^ mice. Seven days after tail vein injection of 20 mg/kg NaIO_3_, H & E staining was performed. The RPE layer is marked with red arrows in all panels. The epithelial RPE monolayer was entirely disrupted, and RPE cells showed a rounded, degenerative phenotype (see arrows) in NaIO_3_-treated groups. Predominant loss of RPE cells, distortion and thinning of ONL, and disorganization of INL and atypical folds in the outer retina was observed in *Ldlr* ^−/−^ mice after NaIO_3_ administration. Representative images from 6–8 animals are shown. Scale bar: 25 µm.

The structural integrity of the retina was assessed via histology 7 days post-NaIO_3_ administration. In the control eyes injected with PBS, all layers of the retina were well organized, and the RPE cell layer was evenly pigmented and could be easily distinguished. However, RPE cells in *Ldlr* ^−/−^ mice appeared larger, rounded, vacuolized, and started to detach from the intact RPE monolayer layer (Figure 5). Displaced cells were evident in the inner plexiform layer (IPL), and the morphology of the INL, outer plexiform layer (OPL), and ONL was abundantly altered along with the development of several atypical folds in the outer retina (Figure 5). As the representative images illustrate, *Ldlr* ^−/−^ mice experienced more severe damage than WT mice, with nearly full degeneration of the cone and rod photoreceptor cells. Most of the photoreceptors and their outer segments were destroyed, coinciding with the functional loss as determined by electroretinography, and the RPE layer was entirely disrupted.

### 3.6. Oxidative Stress Exacerbates Apoptosis in Ldlr ^−/−^ Mouse Retina

**Figure 6:**
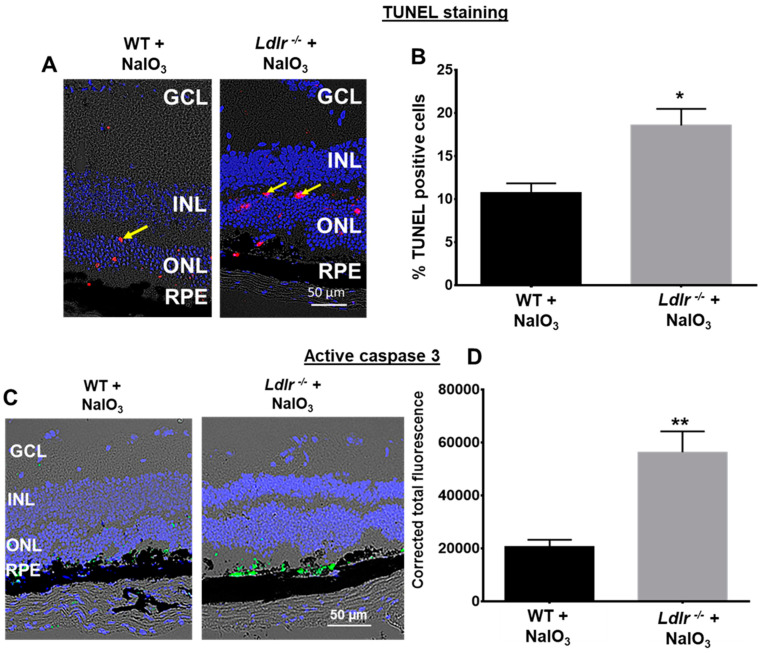
TUNEL and caspase activation in retina 7 days after a single NaIO_3_ (20 mg/kg) treatment in C57BL6 (WT) and *Ldlr* ^−/−^ mice. (**A**). Apoptosis was assessed by TUNEL assay. Apoptotic cells are observed in the ONL, RPE, and choroid. (**B**). Total number of TUNEL positive cells. The percentage of TUNEL positive cells in the *Ldlr* ^−/−^ + NaIO_3_ group was significantly increased. No cell death was observed in PBS-treated WT or *Ldlr* ^−/−^ (not shown). Data are shown as mean ± SEM (*n* = 10, * *p* < 0.0027). (**C**) Activation of caspase-3 in NaIO_3_ challenged mouse retina. (**D**). Quantification revealed that caspase-3 activation was significantly higher in NaIO_3_-treated *Ldlr* ^−/−^ mice when compared with the corresponding stressed WT mice (mean ± SEM, *n* = 6) ** *p* < 0.005.

Retinal functional studies and histology demonstrated significantly increased retinal damage in the *Ldlr* ^−/−^ mice challenged with NaIO_3_. To further explore whether the compromised retinal function relates to apoptosis, we quantified apoptotic cells in the NaIO_3_ challenged retinas. TUNEL staining, which specifically tags fragmented DNA of late apoptosis, revealed that 20 mg/kg NaIO_3_ caused significantly more apoptosis in the *Ldlr* ^−/−^ than the corresponding WT mice treated with NaIO_3_. As shown in Figure 6A,B, TUNEL-positive cells increased in the ONL of the *Ldlr* ^−/−^ mice compared with WT mice after NaIO_3_ treatment, suggestive of damage in the photoreceptor nuclei. Following evidence that LDLR loss worsens cell death in response to a NaIO_3_ challenge, cleaved caspase-3, the primary executor of conventional cell death, was examined in retinal sections. Although there was no evidence of active caspase-3 expression in the control samples (not shown), by day 7 after NaIO_3_ treatment, some cleaved caspase-3 positive cells (green cells in Figure 6B,C) were seen in the RPE and ONL of WT animals. The TUNEL positive cells seen in retinal slices are consistent with the low number of activated caspase-3 signals. However, when compared to comparable WT, the fluorescence intensity for cleaved caspase-3 was considerably higher in *Ldlr* ^−/−^ mice treated with NaIO_3_. Caspase-3 staining was mostly observed only in the RPE cell layer and in a few cells in the photoreceptor layer (Figure 6C). Importantly, we found that *Ldlr* ^−/−^ mice have increased active caspase-3 expression, which could help to explain why they are more susceptible to apoptosis with stress.

### 3.7. LDLR Deficiency Resulted in Lipid Accumulation in the Outer Retina

**Figure 7:**
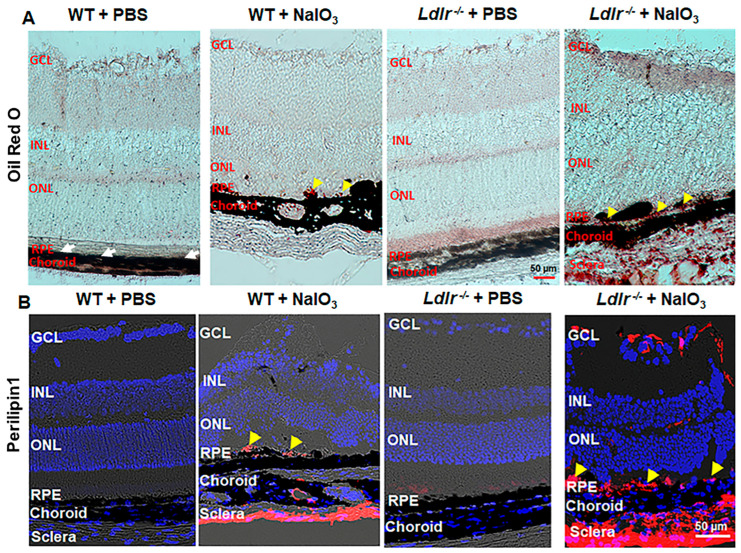
Deficiency of LDLR leads to lipid deposition in the mouse outer retina. Representative images of lipid deposits revealed by neutral lipid staining with Oil Red O (**A**) show more deposits in the *Ldlr* ^−/−^ retina compared to WT challenged with NaIO_3_. Lipid deposits can be seen marked by arrowheads in the RPE. In the WT + PBS, RPE layer is indicated with white arrows. (**B**) Perilipin 1, a protein associated with lipid droplets, was clearly upregulated after being treated with NaIO_3_. The staining was very intense and widely distributed in the *Ldlr* ^−/−^ mice upon NaIO_3_ administration. The yellow arrowheads show perilipin1 expression in the RPE layer. GCL: ganglion cell layer; INL: inner nuclear layer; ONL: outer nuclear layer; RPE: retinal pigment epithelium. Scale bar: 50 µm.

Pathohistological examinations of drusen in human donor eyes have revealed a role for lipids in AMD [26,27]. Given the known role of LDLR in regulating lipid metabolism, we stained retinal cryosections from WT and *Ldlr* ^−/−^ mice challenged with NaIO_3_ with Oil Red O (ORO) staining to evaluate the presence of any hydrophobic lipids, including esterified cholesterol within the retinal layers. ORO revealed that lipid droplets were present in the RPE, BrM, choroid, and sclera of the *Ldlr* ^−/−^ mice; however, lipid staining was sparse in the RPE of the WT mice challenged with NaIO_3_ (Figure 7A). Notably, when compared with WT mice, which did not exhibit any ORO staining, deletion of *Ldlr* markedly increased neutral lipid staining, apparent as red foci, in the RPE, choroid, and sclera from the NaIO_3_-treated mice. Proteins of the perilipin family have been associated with surfaces of lipid droplets and consist of five members [28]. Perilipin 1 expression has been reported in RPE, and tissue samples from the retina and brain [29,30,31]. Limited perilipin 1 staining was seen in the RPE, choroid, and scleral tissues when NaIO_3_ was given to WT mice, but increased perilipin 1 staining was seen in the *Ldlr* ^−/−^ mice when challenged with stress (Figure 7B and Figure S3). These data provide evidence that the genetic deletion of *Ldlr* results in a delayed removal of LDL from tissues, resulting in an enhanced accumulation of neutral lipids with oxidative stress in the RPE and choroidal tissues. However, the origin of these lipids is not clear. Although it could be from delayed removal, it could also be from endogenous synthesis of lipids within these tissues. Consistently, in retinal sections, perilipin-labeled lipid surface signals were more prominently observed in the stressed *Ldlr* ^−/−^ sections.

## 4. Discussion

In this study, we investigated the significance of LDLR in hRPE survival and retinal function in an animal model of dry AMD. We provide several lines of evidence suggesting that LDLR plays an important role in dry AMD. The results obtained in this study demonstrate that the lack of LDL receptor causes: 1. exacerbation of RPE cell death when subjected to oxidative stress, 2. accelerated RPE and photoreceptor degeneration and retinal thinning, 3. impaired retinal functions, 4. augmented apoptosis, and 5. accelerated lipid accumulation in the RPE and choroid.

Intracellular lipid accumulation is dependent on the net balance of lipid transport into and out of the cells [32]. LDL is responsible for the transport of lipids into the cells via the LDLR, whereas HDL participates in the transport of lipids out of the cells [33]. The mechanisms by which HDL removes lipids is called reverse cholesterol transport (RCT) [34]. We believe that lack of regulation of the receptor for LDL may lead to an imbalance in lipid transport resulting in intracellular accumulation of lipids. Additional studies are needed for confirmation of our findings and their link to AMD pathogenesis.

AMD is a complex group of progressive diseases that lead to the degeneration of the macula in older adult individuals and is the leading cause of irreversible blindness in developed nations. The more prevalent dry AMD (also known as atrophic AMD), which affects roughly 8 out of 10 patients with AMD, is characterized by the buildup of sub-RPE deposits (drusen), which are made up of a variety of proteins and lipids that come from both inside and outside the retina. Cholesterol homeostasis of the outer retina, especially as pertains to sterol and lipid uptake and efflux across the RPE, plays a critical role in the pathogenesis of AMD [35]. The LDLR pathway is a negative feedback mechanism that is crucial for maintaining the balance between intracellular and plasma cholesterol levels. Cells accumulate excessive amounts of lipids because of dysregulated LDLR expression [36].

In hRPE cells, LDLR is mostly expressed in the cytoplasmic compartments, and oxidative stress significantly reduced its expression. Prior studies have revealed the presence of the scavenger receptor CD36 and the LDL receptor in RRE cells, which are essential for the internalization of LDL and oxidized LDL (oxLDL) [35,37]. The LDL receptor was localized to the basal RPE cell membrane in monkey retinas by immunohistochemical studies which is consistent with our findings in the polarized RPE monolayer [23]. Based on our data and previous findings, we suggest that plasma LDL appears to enter in and out of the RPE through the basolateral domain from the choriocapillaris endothelium by crossing the BrM.

In our studies on the role of LDLR in RPE cell protection, we silenced Ldlr in ARPE-19 cells since transfection studies in primary RPE cells are challenging, and transfection efficiency varies from donor to donor. The characteristics of ARPE-19 cells were assessed carefully; we verified that ARPE-19 expressed RPE-specific markers BEST1, CRALBP1, and Keratin-18 (Supplemental Figure S1). We used ARPE-19 cells at passage 10 as recommended in a recent report [38].

Although many rodent models for (dry) AMD have been generated, there has not yet been an animal model that can adequately represent the progression of dry AMD disease phenotype. The complex nature of AMD makes phenotypic modeling very difficult. As a result, studies into the etiology and effective therapeutic methods of the disease are significantly hampered by the lack of relevant models. Since NaIO_3_ injection exhibits some phenotypic characteristics like dry AMD, it has been widely employed as a pre-clinical model of geographic atrophy [21,39,40,41]. Similar to what is seen in atrophic AMD, modest doses of NaIO_3_ (corresponding to the dose used in this study) cause RPE cell death, which is followed by a subsequent death of photoreceptors [21,40,41]. The loss of RPE cells not only affects the photoreceptors but also the underlying choriocapillaris. Administration techniques (e.g., intraperitoneal or intravenous), as well as the instability of NaIO_3_ are factors that may be responsible for variations reported in published research [41]. In our model, we were able to confirm the patchy loss of RPE cells as well as the degeneration of the choriocapillaris and photoreceptors. A recent study supported the NaIO_3_ model and demonstrated that intravenous administration of NaIO_3_ provides consistent and reproducible results [40]. This model has already been employed as a testing ground for many experimental RPE-cell-based therapies as well as bone marrow transplantation [40,42,43,44,45].

The interaction between oxidative stress and inflammation has been the subject of numerous investigations [3,46]. A number of preclinical investigations have shown that oxidative stress stimulates the initiation and amplification of inflammatory pathways in a wide range of cell types in the retina and choroid [47,48,49]. Recent research has also shown that the NaIO_3_ model modulates soluble immune proteins, complement proteins, and cytokines/chemokines in a time-dependent manner, supporting the idea that this model could be used to mimic retinal inflammatory sequences [41,50]. *Ldlr* ^−/−^ mice fed with high-fat diet for 12 weeks had increased oxidized lipids and inflammatory markers and caused pulmonary hypertension [51], suggesting a role for LDLR and oxidized lipids in the initiation of diseases. However, in-depth research is required to specify how LDLR and oxidized lipids interact in our model of dry AMD.

We reported previously that NaIO_3_ treatment caused significant retinal function impairment in WT mice [22]. However, whether LDLR deficiency accentuates altered retinal dysfunction has not been hitherto addressed in detail. The LDL receptor knockout model serves as an established model for atherosclerotic pathomechanisms because of the opportunity to induce elevated plasma total cholesterol levels [52]. In addition, increased triglycerides were observed in different tissues of *Ldlr* ^−/−^ mice used in other disease models such as non-alcoholic fatty liver disease [53], atherosclerosis [54,55], *Ldlr* ^−/−^ Leiden infertility [56] and pulmonary hypertension [57]. Due to the association between the existence of such lipid deposits and AMD and the fact that these particles bind ORO and include esterified cholesterol and triglycerides [4,5,6], we utilized the *Ldlr* ^−/−^ model to investigate its function in dry AMD. Lipids are important components of the retina and make up to 50% of the dry matter in rod outer segment membranes [4,58]. Thus, dysregulation of LDLR could cause a change in fatty acid status and may be etiologically involved in the reduction of the ERG response seen in our study. We demonstrated that stressed *Ldlr* ^−/−^ mice exhibited an increased accumulation of lipid droplets in the outer retina, supporting the hypothesis that disruption of lipid homeostasis may influence retinal function. In eyes of apoB100, *Ldlr* ^−/−^ mice, buildup of esterified cholesterol was also observed at the basement of the retinal pigment epithelium [59]. Similar findings were observed in apoE-deficient mice, which led to a significant reduction in the amplitudes of the a- and b-waves [59,60]. Increased intracellular lipid deposition were also observed in 24-month-old male *Ldlr* ^−/−^ mice [24]. *Ldlr* ^−/−^ mice exhibited thickening of the BrM with accumulation of lipid particles, which is further increased after fat intake due to elevated blood lipid levels [61]. Although the exact mechanism of retinal degeneration in *Ldlr* ^−/−^ mice is unknown, it is possible that the absence of LDLR may inhibit the transfer of cholesterol from circulating LDL into RPE and other neural retinal cells, causing cholesterol buildup and this likely leads to photoreceptor loss and retinal dysfunction.

Multiple sources of evidence refer to RPE death followed by photoreceptor loss by apoptosis as the final stage leading to irreversible blindness in retinal pathologies such as AMD and retinal detachment [62,63]. The lipid metabolism–cholesterol pathway appeared genetically in candidate gene and later GWAS investigations, and it has long been linked to AMD [64,65,66,67]. Several mechanisms may be involved as to why decreased LDLR levels result in greater susceptibility to cell death. According to our in vitro results, the activation of caspase-3/7 over time and the death of RPE cells was greatly accelerated by LDLR silencing followed by stress exposure. With NaIO_3_ administration in the *Ldlr* ^−/−^ mice, we observed notable apoptotic cell death and caspase-3 activation. Cholesterol has been linked to several reports that suggest it may cause cell death [24,68,69]. Even with a normal diet, the plasma total cholesterol of *Ldlr* ^−/−^ mice is higher because they cannot adequately incorporate plasma cholesterol into their body cells [70,71]. Similar results have been shown in an animal model of pulmonary fibrosis and familial hypercholesterolemia, where aberrant LDL-LDLR metabolism promotes apoptosis and contributes to the dysfunction of endothelial and epithelial cells [72,73]. Distinguishing the precise pathways which causes the apoptosis, through either the ROS produced by NaIO_3_ or cholesterol dyshomeostasis, or a combination of the two, is of interest but will need additional work.

## 5. Conclusions

In summary, we demonstrated that LDLR deficiency caused caspase-3/7 activation and increased cell death in RPE. NaIO_3_-treated *Ldlr* ^−/−^ mice displayed markedly dysregulated retinal function as significant thinning of the retina and reduced ERG. Furthermore, stressed *Ldlr* ^−/−^ mice showed increased lipid accumulation and susceptibility to apoptotic cell death. The *Ldlr* ^−/−^ mouse model is thus particularly valuable for understanding the relationships between lipid metabolism and oxidative stress in the context of AMD. The *Ldlr* ^−/−^ mouse model may be considered a suitable platform for evaluating therapeutic approaches for dry AMD.

## Data Availability

Not applicable.

## Supplementary Materials

The following supporting information can be downloaded at: https://www.mdpi.com/article/10.3390/cells12010043/s1, Figure S1: RT-PCR of RPE specific biomarkers in cultured ARPE-19 cells; Figure S2: Localization of LDLR in polarized RPE monolayer; Figure S3: Quantification of perilipin 1 fluorescence intensity; Table S1: Primers used in this study.
