# Supplementary material for: Oxidative Stress and Lipid Accumulation Augments Cell Death in LDLR-Deficient RPE Cells and Ldlr −/− Mice
Source: Cells. 2022 Dec 22;12(1):43. doi: 10.3390/cells12010043 (PMC9818299; doi:10.3390/cells12010043)
Supplement: Supplementary file 1 [file cells-12-00043-s001.zip › cells-1996480-supplementary.pdf]

Supplementary Figure S1

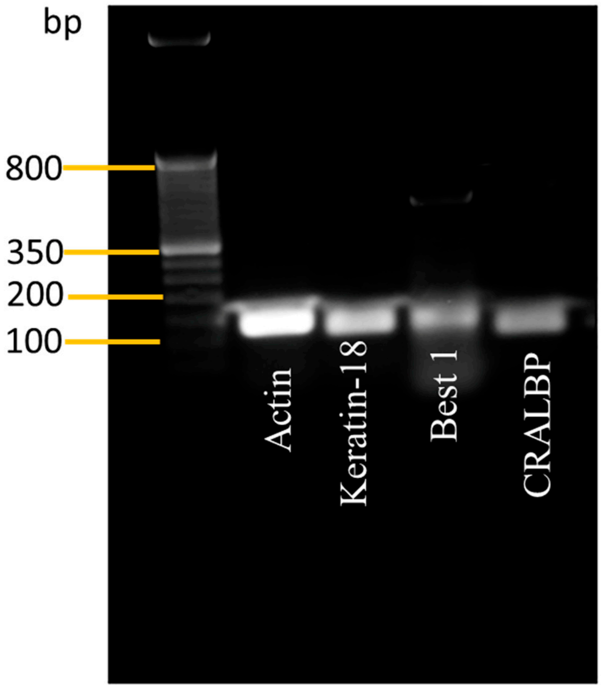

RT-PCR of RPE specific biomarkers in cultured ARPE-19 cells. ARPE-19 cells expressed epithelial markers Keratin-18, Bestrophin1 (Best1), and Cellular retinaldehyde–binding protein (CRALBP). Actin was used as loading control.

Supplementary Figure S2

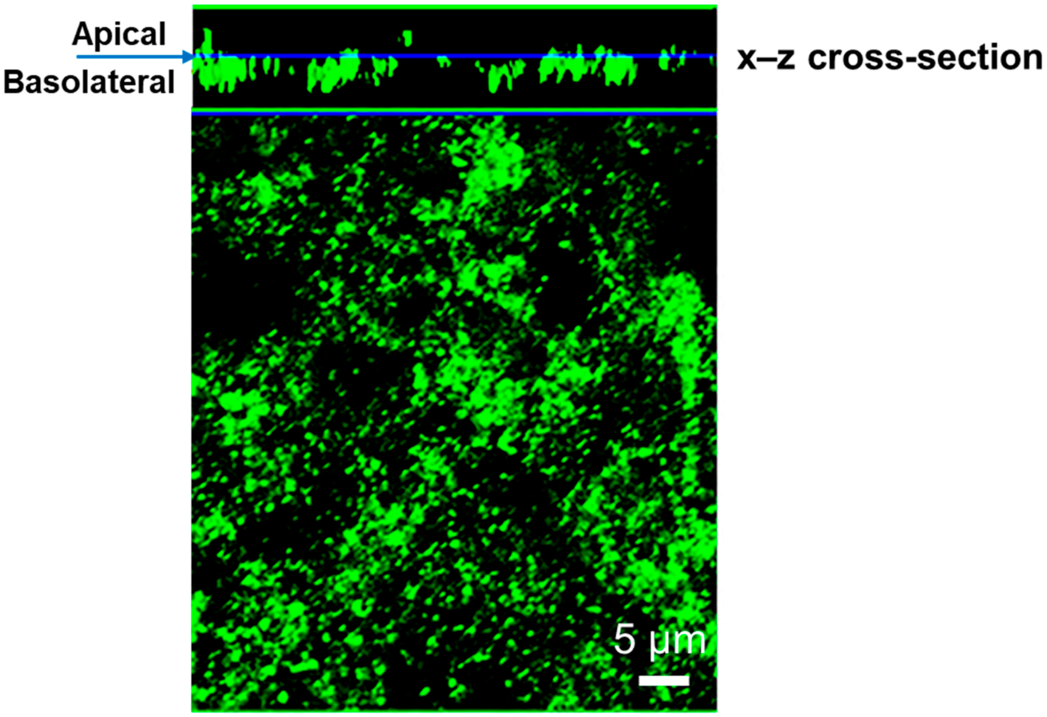

Localization of LDLR in polarized RPE monolayer. Highly differentiated fetal human primary RPE cells were cultured on Transwell filters as described [15]. Polarized monolayers were fixed in 4% PFA, blocked, and incubated overnight with LDLR antibody (1:100 dilution, # PA5-22976, Invitrogen). After incubation with FITC-conjugated secondary antibody (1:100 dilution, Vector Laboratory), cells were imaged using a confocal microscope (LSM 710). Basolateral localization of LDLR is indicated by an arrow.

Supplementary Figure S3

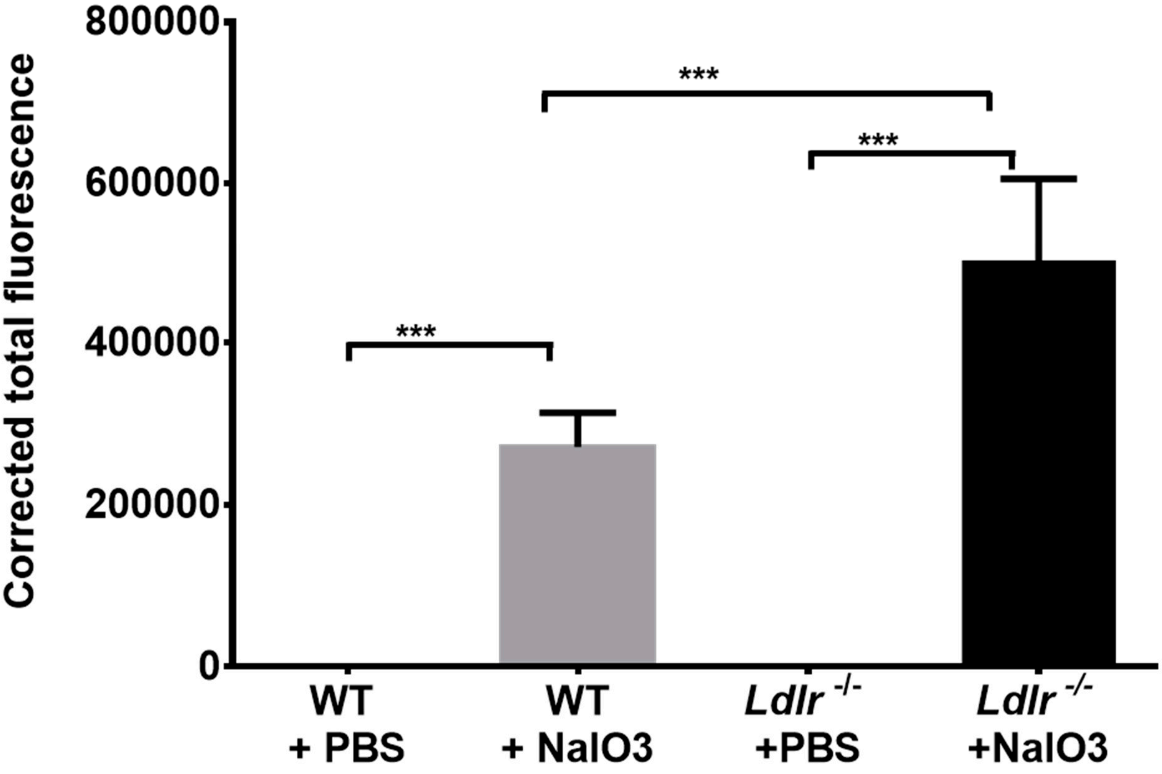

Quantification of perilipin1 fluorescence intensity. ImageJ was used to assess the fluorescence intensity, and the results showed that perilipin 1 levels in NaIO3-treated *Ldlr* $^{-/-}$ mice were considerably greater than those in the correspondingly stressed WT mice. There was no detectable staining in the *Ldlr* $^{-/-}$ + PBS group or the WT+ PBS groups. (mean ± SEM, n= 6) *** $p<0.001$.

Table S1. List of primers used in this study

Target Gene	Primer sequences
BEST1-F	5'-TGC CAA CCT GTC AAT GAA GGC G-3'
BEST1-R	5'-TCC AGT CGT AGG CAT ACA GGT G-3'
CRALBP-F	5'-GGC AAA GTC AAG AAA TCA CCT T-3'
CRALBP-R	5'-AGC CAT TGA TTT GAG TTT CCT C-3'
Cytokeratin 18 -F	5'-GCT GGA AGA TGG CGA GGA CTT T-3'
Cytokeratin 18 -R	5'-TGG TCT CAG ACA CCA CTT TGC C-3'
Actin-F	5'-CAC CAT TGG CAA TGA GCG GTT C-3'
Actin-R	5'-AGG TCT TTG CGG ATG TCC ACG T-3'

BEST-1: Bestrophin-1; CRALBP: Cellular retinaldehyde–binding protein
